# Novel concept for the healthy population influencing factors

**DOI:** 10.3389/fpubh.2024.1387255

**Published:** 2024-12-10

**Authors:** Yuhao Shen, Jichao Wang, Lihua Ma, Huizhe Yan

**Affiliations:** ^1^Business School, Inner Mongolia University of Finance and Economics, Hohhot, China; ^2^School of International Education, Anyang Institute of Technology, Anyang, China; ^3^School of Management Engineering and Business, Hebei University of Engineering, Handan, China

**Keywords:** migrant population, health determinants, machine learning algorithms, public health policy, predictive modeling

## Abstract

In the rapid urbanization process in China, due to reasons such as employment, education, and family reunification, the number of mobile population without registered residence in the local area has increased significantly. By 2020, the group had a population of 276 million, accounting for over 20% of the total population, making significant contributions to urban economic development and resource optimization. However, the health status of migrant populations is affected by unique issues such as occupational risks and socio-economic disparities, which play an important role in personal welfare, social stability, and sustainable economic growth. The deterioration of the health of the floating population will lead to a decrease in productivity, an increase in medical expenses, and an increase in pressure on the public health system. In order to analyze and predict the main elements affecting the well-being of transient population, this study uses advanced machine learning algorithms such as principal component analysis, backpropagation (BP) neural networks, community analysis, random forest models, etc. Principal component analysis will identify and extract the most important variables that affect the health status of mobile populations. The BP neural network models the nonlinear interaction between health determinants and health outcomes. Community analysis divides the floating population into different health records and promotes targeted intervention measures. The random forest model improves the accuracy and universality of predictions. The insights generated by these models will help develop health policies and intervention policies to improve the health status of mobile populations, narrow disparities, and promote social and economic stability. Integrating data-driven methods and emphasizing a shift towards correct, effective, and impactful public health management provides a robust framework for understanding and addressing the complex health issues faced by mobile populations.

## 1 Introduction

Floating population refers to individuals who have moved from their habitation to other places due to employment, education, or family reunion, but do not have a local registered place of residence. In the rapid urbanization process in China, this group continues to expand and become an important component of social and economic development. According to data from the National Bureau of Statistics, China’s floating population is expected to reach 276 million by 2020, accounting for over 20% of the total population. This means that one in every five people is a floating population. In 2023, the number of inter-regional movements in China was 61.25 billion, an increase of 30.9% from the previous year. The floating population plays an important role in the labor market, providing a large amount of labor and promoting urban economic development and construction (1). Has made important contributions to the rational allocation and optimization of urban and rural resources. The health condition of the floating population not only affects the quality of life and happiness of individuals, but also has a profound impact on social stability and sustainable economic development. Firstly, mobile populations with poor health conditions may face issues such as low work efficiency and high medical costs, which may have a negative impact on family economic conditions and overall quality of life (2). Secondly, the health issues of the floating population impose a burden on the urban public health system, increasing the demand and pressure for medical resources. In addition, improving the health status of the floating population can enhance work and production efficiency, promote economic growth and social development (3).

The purpose of this study is to use advanced machine learning algorithms to analyze the principal considerations affecting the well-being of floating populations, develop predictive models, and guide the formulation of relevant policies. In particular, Principal Component Analysis (PCA) plays an important role in reducing the hierarchy of the dataset while maintaining basic information. This technology helps to identify and extract the most important variables that affect the health status of mobile populations. PCA improves the efficiency and interpretability of subsequent machine learning models by transforming the original related variables into smaller independent principal component subsets. The study will use principal component analysis, inverse wave neural networks, community analysis, and random forest models. These methods were chosen because they possess typical characteristics of public health data, complex nonlinear relationships, and robustness in handling large-scale datasets. Especially, BP neural networks are used to model the nonlinear interactions between different health determinants and the health outcomes of mobile populations. It is particularly effective in capturing complex relationships that traditional statistical methods may not be able to display (4). Community analysis categorizes the floating population into multiple types based on risk factors related to health records. This classification helps identify vulnerable subgroups and adjust specific intervention measures to meet their needs. The random forest model is used to improve the accuracy and universality of health outcome prediction. By summarizing the results of principal component analysis, the random forest algorithm can reduce the risk of over matching and improve the reliability of prediction models. Therefore, the insights gained from these machine learning models will provide scientific basis for developing targeted health policies and intervention policies. By understanding the main factors affecting the health of mobile populations, decision-makers can design more effective public health strategies, improve the health status of mobile populations, narrow health disparities, and promote social and economic stability (5).

The health issues of mobile populations have had a profound impact on individual health and broader social development. Due to factors such as occupational risks, inadequate medical services, and socio-economic disparities, mobile populations often face unique health problems. Improving their health status is not only important for improving their quality of life, but also for ensuring social stability and economic productivity (6). A healthy floating population has an effective contribution to the labor market, supports family economy, and is likely to smoothly integrate into the new community. Therefore, meeting the health needs of this group is crucial for promoting inclusivity and sustainable development (7). Meanwhile, applying data-driven methods in public health research is crucial for further understanding complex health issues and developing effective intervention measures. Principal Component Analysis (PCA), BP neural networks, cluster analysis, random forests, and other machine learning algorithms provide powerful tools for analyzing large and complex datasets. These methods can discover hidden patterns, identify the main determinants of health outcomes, and accurately predict future health trends. By utilizing these advanced analytical techniques, researchers can establish evidence-based policies and generate practical insights to guide targeted health interventions. In the context of this study, data-driven methods provide a robust framework for analyzing complex elements influencing the well-being of floating populations. The predictive model developed in this study not only enhances our understanding of the fitness trends of floating populations, but also guides the development of targeted public wellness strategies. Ultimately, integrating machine learning into public health research will transform into more precise, efficient, and influential health management. This study will focus on the factors that affect the health of China’s floating population, but similar issues also exist worldwide (8). For example, in Europe, the health outcomes of mobile populations are influenced by socio-economic status, access to medical services, and comprehensive policies. In the United States, factors such as immigration status, mental health, and employment conditions have a significant impact on the health of mobile populations. By comparing these international backgrounds, we can better understand the challenges that mobile populations typically face, especially in literature research where we can explore these challenges more specifically.

**Figure 1:**
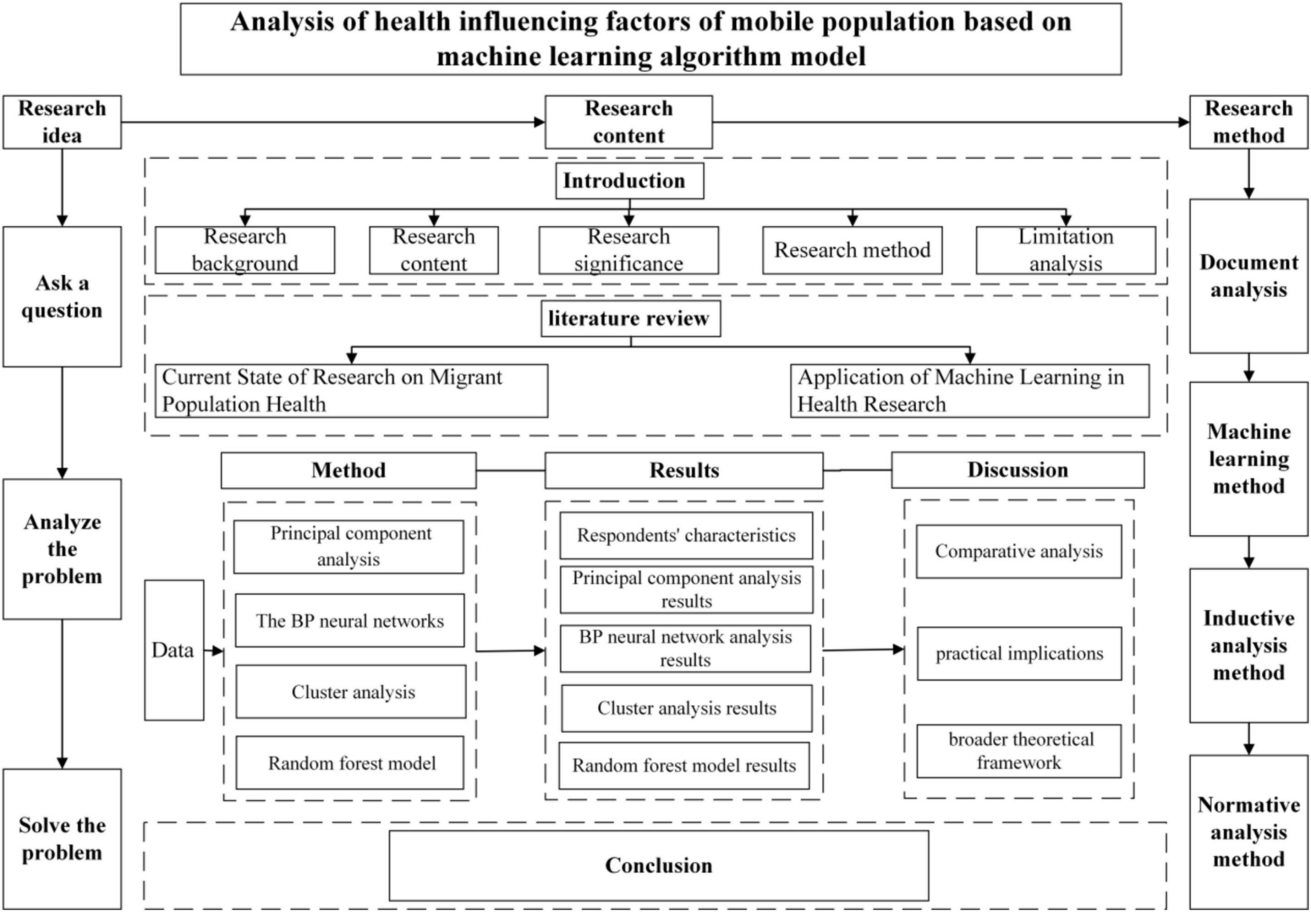
Theoretical framework diagram.

In order to clarify the relationship between the research results and the theoretical framework more clearly, we provide a theoretical framework chart in Figure 1.

The methodology and data analysis of this study are very rigorous and comprehensive, but there are still several limitations that need to be discussed. Firstly, the research scope is limited by available data and cannot capture all relevant health determinants, nor can it reflect the dynamic health characteristics of mobile populations affected by changes in socio-economic conditions. Secondly, potential biases in data collection such as insufficient reporting of health issues and incorrect recording of socio-economic details can affect the appropriateness of the results. Floating populations may also face language barriers or insufficient trust in data collectors, which may lead to data bias. Recognizing these limitations can provide a more detailed understanding of the scope and potential applications of the research (9), address these challenging issues, and indicate future research directions for improving the reliability of research results on the health of mobile populations.

By utilizing the advantages of multiple machine learning modes, this study not only enhances our understanding of health determinants for different population groups, but also lays the foundation for future research to improve health outcomes through data foundation and predictive analysis. Integrating these methods into health research can greatly promote the development of more effective public health strategies and resource allocation, ultimately improving the healthy lives of mobile and regional populations.

## 2 Literature review

### 2.1 Current state of research on migrant population health

In recent years, the health issues of mobile populations have received much attention in the field of public health. With the acceleration of globalization and urbanization, more and more people are moving between cities, rural areas, and different regions due to reasons such as occupation, education, and living conditions. However, while enjoying economic development, the holistic health situations of the transient population is relatively poor due to insufficient health protection and difficulty in accessing medical services (10, 55). The existing literature mainly explores the health status of mobile populations from two perspectives: technical research and mechanical research. Technical research mainly uses cross-sectional data to illustrate and compare the health status of mobile populations. For example, He et al. (11) found from the dynamic monitoring data of China’s floating population that the floating population lagged behind the non-floating population in terms of health self-assessment, chronic disease incidence rate and mental health. The focus of mechanical research is to explore various factors such as socio-economic status, accessibility to medical services, social support, and the underlying mechanisms that affect health (12). The purpose of these studies is to understand the impact of these factors on health outcomes and provide potential intervention plans to improve the health of mobile populations.

The well-being status of floating populations is affected by kinds of elements, among which socio-economic factors, accessibility to medical services, lifestyle habits, and environmental factors are the most studied aspects. Firstly, from the perspective of socio-economic factors, socio-economic status has a significant impact on the health of mobile populations. Factors such as income level, education level, and occupational type not only directly affect personal health, but also indirectly affect it through media factors such as medical service utilization, living environment, and health actions (13). Low income mobile populations are more vulnerable to health risks, such as malnutrition, insufficient awareness of disease prevention, and difficulty accessing medical services (14). Research has shown that migrant populations with lower levels of education lack health knowledge and disease prevention awareness, which increases the risk of health problems (15). Recent studies have further confirmed the profound impact of socio-economic factors on the health of mobile populations. For example, Hu et al. (16) found a significant relationship between the income level of mobile populations and their self-assessment of health, with higher income levels representing better health status. In addition, education level has been proven to be an important factor affecting health, and higher education levels can help improve the ability to understand health articles and prevent diseases (17). Secondly, in terms of medical service accessibility, medical service accessibility is an important factor affecting the health of mobile populations (18). There are many situations where resident registration restrictions and mobility make it difficult for mobile populations to access stable medical services. Difficulty in accessing medical services not only affects health management, but also increases the cost of treating diseases. Jorg and Haldimann (19) pointed out that the low penetration rate of health insurance is the main reason for the low utilization rate of medical services among mobile populations. Improving the prevalence of health insurance and reducing medical costs is an important method to improve the health status of mobile populations. Other studies have shown that the construction and promotion of regional health service institutions have a positive impact on improving the accessibility of medical services for mobile populations (20, 21). Thirdly, in terms of lifestyle factors, lifestyle habits are an important factor affecting the health of mobile populations (22). The high work pressure and harsh living environment make it easy for mobile populations to develop unhealthy habits such as smoking, drinking, irregular sleep patterns, and insufficient exercise (23). These bad habits not only directly harm health, but also increase the risk of chronic diseases and have long-term negative effects on health (24). Recent studies have further revealed the impact of unhealthy lifestyle habits on the health of mobile populations. Lin et al. (25) found that long-term work pressure and harsh living conditions are important reasons for unhealthy living habits among migrant populations. In addition, the dietary habits of mobile populations, such as imbalanced diets and intake of high-fat and high sugar foods, can increase the risk of nutritional deficiencies and chronic diseases. Health education and lifestyle interventions are considered important measures to improve the health status of mobile populations (26). Fourthly, in terms of social support, it plays a buffering role in the health of the floating population. Social assistance systems, including family assistance, colleague assistance, and community assistance, can alleviate the pressure faced by mobile populations in adapting to new environments and addressing health issues. Research has shown that strong social support will greatly improve the psychological and overall health of mobile populations (27). Chawla et al. (28) emphasized the importance of social assistance for the health of mobile populations. They found that support from families and communities has a positive impact on the mental health of migrant populations. In addition, establishing good colleague relationships and receiving support in the workplace can also help improve the health status of mobile populations (29).

In summary, the health status of floating population is subjected to multiple factors such as socio-economic factors, accessibility to medical services, lifestyle habits, and social support. These factors interact with each other to determine the well-being degree of the floating population. Therefore, in order to address the health issues of the transient population, it is necessary to consider these impacts and adopt multi-level and multidisciplinary intervention measures to improve the general health condition of the floating population.

### 2.2 Application of machine learning in health research

In recent years, the application of machine learning technology in health research has rapidly increased, becoming an important tool for analyzing health influencing factors. Compared with traditional statistical methods, machine learning can handle large and complex datasets and identify nonlinear relationships and patterns hidden within the data. The following is an example study of using machine learning to analyze health influencing factors. Chronic diseases are an important factor affecting the health of the world’s population, and early prediction and intervention are crucial to improve the health status of patients with chronic diseases. Machine learning algorithms can analyze large-scale health data, such as electronic health records (EHRs), to effectively predict the risk of chronic diseases. Parthiban et al. (30) used machine learning algorithms such as random forest and gradient elevator to analyze and predict the health data of diabetes patients. The results show that this algorithm can accurately predict the occurrence of diabetes, and identify the main health influencing factors such as age, body mass index (BMI) and blood sugar value. Psychological health issues are the main public health issues in modern society. By analyzing social media data, mental health questionnaires, and biosensor data, machine learning techniques can effectively evaluate an individual’s mental health status. Wang et al. (31) used natural language processing (NLP) techniques and machine learning algorithms such as support vector machines (SVM) to analyze text data on social media platforms and predict user depression and anxiety states. Research has shown that machine learning algorithms have high accuracy in predicting and identifying mental health issues. Public health monitoring is important for controlling the spread of infectious diseases. By analyzing real-time data, machine running technology can improve the efficiency and accuracy of public health monitoring. Sun et al. (32) used deep learning algorithms to comprehensively analyze public health data and geographic information system (GIS) data to predict and monitor the spread of influenza. Research has shown that deep learning algorithms can accurately predict the time and location of influenza occurrence, providing scientific basis for public health interventions. Machine learning can not only analyze a single type of health influencing factor, but also multiple factors to reveal complex mechanisms that affect health status. Chen et al. (33) combined electronic health records, genetic data, and environmental exposure data to analyze the impact of multiple factors on cardiovascular disease using collective learning algorithms. Research has found that ensemble learning algorithms can effectively integrate multi-source data and identify the main health influencing factors and their interactions. Health risk assessment is the foundation for developing personalized health management plans. Machine learning algorithms can comprehensively analyze personal health data and provide accurate health risk assessments. Huang et al. (34) used machine learning algorithms such as beige networks and logistic regression to analyze and evaluate health data of cancer patients. The results indicate that these algorithms can accurately evaluate individual health risks and provide scientific basis for personalized health management.

In short, the application of machine learning technology in health research is becoming increasingly widespread, providing powerful tools for analyzing health influencing factors and health management. In the future, with the enrichment of data resources and continuous optimization of algorithms, the application prospects of machine learning in health research will be even broader (35).

## 3 Data and method

### 3.1 Data source

This study used data from 31 provinces in China in 2018 to develop an indicator system for measuring the health status of the floating population. These data mainly come from the large-scale national sampling survey conducted by the National Health Commission – China Mobile Population Dynamic Monitoring Survey (CMDS). CMDS covers 31 provinces and Xinjiang Production and Construction Corps, where the floating population is highly concentrated. This survey uses proportional sampling (PPS) and targets individuals aged 15 and above who have resided in their catchment area for more than a month and have not registered residence. These data include basic information on the health status, participation in medical insurance, gender, age, education level, marital status, employment status, household registration, mobility scope, reasons for mobility, income, health education, and health records and other fields. As the focus of this study is on the health status of the floating population, the sample is limited to people aged 16 to 60 and further filtered. After data processing, 89,346 samples were ultimately selected from 31 provinces. At the same time, we recognize that the differences in economic development levels in different regions of China may affect individuals’ health status and immigration patterns. Therefore, we incorporated regional economic indicators into the model to monitor regional differences and ensure more accurate analysis of health factors.

Specifically, the CMDS survey collects extensive data through questionnaires to better understand the living and health conditions of the floating population. The basic information section includes demographic characteristics such as gender, age, race, education level, and family status. The section on health status mainly includes diagnostic information provided during hospital examinations or treatments. The section on health insurance refers to whether an individual has participated in various health insurances, such as the basic health insurance for urban workers, the new rural cooperative health insurance, and the basic health insurance for urban residents. In addition, the survey also collected information on the employment situation of the floating population, including their employment status. Family registration information includes the type of family registration system and place of residence. The section on the scope and reasons for migration provides a detailed record of the frequency, distance, and main reasons for migration, such as employment, education, and family reunification. The section on income and health education identifies the income levels of individuals and their families, as well as the pathways through which they acquire health knowledge.

We recognize that the differences in economic development levels between different regions of China may affect individual health status and mobility patterns. Therefore, in the subsequent empirical analysis, we will further analyze and discuss the health status of the floating population from the perspective of regional differences in characteristics. This helps to better understand the differences in health status and migration patterns between different regions, and provides more comprehensive background information. Here, for the division of urban and rural hukou, the respondents with agricultural hukou are considered as rural registered residence, and those with non-agricultural hukou are considered as urban registered residence families. Since the Migrant Population Questionnaire (Eight Cities) (Volume C) and the registered residence Population Questionnaire (Eight Cities) (Volume D) are selected from eight representative cities (states and districts) in the sampling framework established on the basis of the 2016 annual report data of the National Migrant Population Information System, using the stratified, multi-stage, proportional PPS sampling method, this paper will analyze these data to better understand the health status of China’s migrant population and its influencing factors, and provide a scientific basis for policy formulation. For example, we can study how participating in health insurance affects an individual’s health status, or the differences in health status between different ages, genders, education levels, and family backgrounds. In addition, we can study the differences in health status between economically developed and underdeveloped regions, and how these differences affect the migration patterns of the floating population. The region is divided into eastern cities (Guangdong, Jiangsu, Shandong), central cities (Hunan, Shanxi, Henan), and western cities (Chongqing, Yunnan, Guangxi).

Here is a detailed description of each feature: Firstly, health status. Among them, a health value of 3 indicates that the individual is in a good state of health; The basic health value is 2, indicating that the individual is in a state of basic health with minor health problems; An unhealthy value of 1 indicates that the individual’s health condition is poor and there are serious health problems. Secondly, the participation in medical insurance. The participation value is 1; The non-participation value is 2. Thirdly, gender. The value for males is 1; The value for females is 2. Fourthly, age. The value for 15–30 years old is 1; The value for ages 31–45 is 2; The value for ages 46–60 is 3; The value for individuals aged 61 and above is 4. Fifth, education level. Illiteracy has a value of 1; The value for primary school is 2; The value for junior high school is 3; The value for high school is 4; The university has a value of 5. Sixth, marital status. Married with a value of 1; The value for unmarried individuals is 2. Seventh, employment status. The on-the-job value is 1; The unemployment value is 2. Eighth, household registration. The urban household registration value is 1; The rural household registration value is 2. Ninth, the scope of mobility. A cross city value of 1 indicates individual mobility between cities; A cross provincial value of 2 indicates individual mobility between provinces; A cross national value of 3 indicates that individuals move between countries. Tenth, reasons for mobility. The value of work reason is 1; The value for other reasons is 2; The value for family reasons is 3. Eleventh, income. The minimum income (below the 20th percentile) is set to 1; The value for lower income (20–39th percentile) is 2; The value of middle-income (40–59 percentile) is 3; The value of higher income (60–79 percentile) is 4; The highest income (above the 80th percentile) is set at 5. Twelfth, health education. The value is 1, indicating that the individual has received health education; A value of 2 indicates that the individual has not received health education. Thirteenth, health records. A value of 1 indicates that the individual has a health record; A value of 2 indicates that the individual does not have a health record.

In addition, relevant studies using the existing China Mobile Population Dynamic Monitoring Data (CMDS) database were studied and listed to support the rationality and background of the research results. These references include Sarkar et al. (36)’s study on the relationship between mobility patterns and mental health outcomes of Chinese immigrant workers, providing insights into the psychological challenges faced by the group (62). Wang et al. (23) provided a comprehensive analysis of the access and use of medical services by studying the utilization and determining factors of medical services for mobile populations.

This study is based on hospital certification and determines the health status of both mobile and local populations. This is a widely recognized standard. If relying solely on self-evaluation, there may be limitations such as subjective bias and cognitive errors. Therefore, using hospital certification as an indicator of health status can improve the reliability and scientific rigor of research results (37).

Due to the differences in the size and characteristics of the selected indicators, including positive and negative indicators, in order to eliminate the differences in indicators, improve the stability and accuracy of the model, the indicators are standardized. The standardization methods used are as follows.

The formula for the standardization of positive indicators as seen in Equation (1):


(1) $$zx_{i}=\frac{x_{i}-\min x_{i}}{\max x_{i}-\min x_{i}}$$

The formula for the standardization of negative indicators as seen in Equation (2):


(2) $$zx_{i}=\frac{\max x_{i}-x_{i}}{\max x_{i}-\min x_{i}}$$

*zxi* represents the standardized value of the *i* indicator.

### 3.2 Method

#### 3.2.1 Principal component analysis

Principal Component Analysis (PCA) is a statistical method that can maintain most of the variability by reducing the dimensions of the dataset. PCA achieves this goal by transforming the original variables into a new, independent set of variables. The arrangement order of these main components maintains most of the variability of the original dataset. PCA is widely used for exploratory data analysis, making predictive models more efficient and less adaptable.

Due to the special nature of questionnaires and answer options, the original dataset consists of sample variables for specific answers to specific questions, resulting in differences in unit selection and dimensional scales (38). In order to improve the comparability of data indicators, necessary preprocessing was performed on the raw data before conducting empirical analysis. The health condition of the floating population is an pointer of various degrees, and standardization is required before conducting principal component analysis on the selected indicators. In addition, the reverse indicators have undergone necessary transformations, uniformly reflecting the degree of progress from low to high.

In this study, the use of PCA can effectively perform data preprocessing and reduce dimensions, thereby improving the performance of machine learning models. It simplifies the dataset while retaining the main information, thus more effectively and accurately analyzing the health impacts of mobile populations (39).

#### 3.2.2 The BP algorithm

The BP algorithm is currently one of the most important and widely used algorithms in artificial neural networks. BP neural network is a leading, multi-layer, non-recurrent neural network that can complete multiple mappings from input to output. It adopts the error backwave algorithm, and the most basic idea is inclined descent. This is a skewed exploration method used to minimize the average squared error between the actual output of the network and the expected output. Information enters through the input layer, passes through the intermediate layer (i.e., the hidden layer), and enters the output layer to generate output results. The optimization goal of BP neural networks is to minimize empirical risk and is usually suitable for large-scale simulations. In this paper, due to the large sample size of mobile population monitoring data, the BP model was chosen. The specific training process is as follows:

1. Network Initialization: First, determine the input and output sequences (X, Y) in the system, the number of nodes nnn in the network’s input layer, and the number of nodes m in the hidden layer. Then, initialize the connection weights wij and wjk between the input layer, hidden layer, and output layer, initialize the hidden layer threshold a and the output layer threshold b, and set the learning rate and activation function (Sigmoid function). As seen in Equation (3).
(3) $$f\left(x\right)=\frac{1}{1+e^{-x}}$$
2. Calculation of the Hidden Layer Output. Based on the input vector X, the connection weights wij between the input layer and the hidden layer, and the hidden layer threshold a, the hidden layer output matrix H is calculated. As seen in Equation (4).
(4) $$H_{j}=f\left(\overset{n}{\underset{i=1}{\sum }}w_{ij}x_{i}-a_{j}\right)j=1,2,\ldots ,l$$
  where *i* is the number of hidden nodes, and f is the activation function of the hidden layer.
3. Calculation of the Output Layer. Based on the hidden layer output vector H, the connection weights wjk, and the threshold b, the predicted output vector Y of the BP neural network is calculated. As seen in Equation (5).
(5) $$H_{j}=f\left(\overset{l}{\underset{j=1}{\sum }}w_{j}x_{jk}-a_{k}\right)k=1,2,\ldots ,m$$
4. Calculation of Error. Based on the network’s predicted output vector Y and the desired output vector O, the network prediction error e is calculated. As seen in Equation (6).
(6) $$e_{k}=O_{k}-Y_{k}k=1,2,\ldots ,m$$
5. Update of Weights. Based on the network prediction error e, the connection weights w_ij_ and w_jk_ are updated.where *η* is the learning rate. As seen in Equations (7, 8).
(7) $$w_{ij}=w_{ij}+\eta H_{j}\left(1-H_{j}\right)x\left(i\right)\overset{m}{\underset{k=1}{\sum }}w_{jk}e_{k}i=1,2,\ldots ,n;j=1,2,\ldots ,l$$
(8) $$w_{jk}=w_{jk}+\eta H_{j}e_{k}j=1,2,\ldots ,l;k=1,2,\ldots ,m$$
6. Update of Thresholds. Based on the network prediction error e, the node thresholds a and b are updated. As seen in Equations (9, 10).
(9) $$a_{j}=a_{j}-\eta H_{j}\left(1-H_{j}\right)\overset{m}{\underset{k=1}{\sum }}w_{jk}e_{k}j=1,2,\ldots ,l$$
(10) $$b_{k}=b_{k}+e_{k}k=1,2,\ldots ,m$$
7. If the error has not yet reached the desired level, repeat steps 2 through 6.

To address the dimensional differences and the variance between positive and negative indicators, standardization of the selected indicators is essential. This process aims to eliminate discrepancies among indicators and enhance the stability and accuracy of the model. The standardization method is as follows:

The standardization formula for positive indicators as seen in Equation (11):


(11) $$zx=i\frac{x_{i}-\min x_{i}}{\max x_{i}-\min x_{i}}$$

The standardization formula for negative indicators as seen in Equation (12):


(12) $$zx=i\frac{\max x_{i}-x_{i}}{\max x_{i}-\min x_{i}}$$

#### 3.2.3 Cluster analysis

Cluster analysis is a statistical method that groups similar objects into their corresponding categories or clusters. The goal of this technology is to ensure that the similarity between objects in the same community is higher than that between objects in other communities. Hierarchical community analysis is the process of merging small communities into larger ones or dividing larger communities into smaller ones to construct a specific type of community structure.

In order to clarify the relationship between multiple variables that affect the health status of mobile populations, this study used stratified communization. The specific stage includes processing the dataset during the data preparation phase and performing necessary transformations to adapt to the analysis. Afterwards, use SPSS software for hierarchical clustering and use the average connection standard. Finally, perform a tree diagram analysis to describe and identify different clusters of variables.

Cluster analysis can help identify relevant variable groups, understand which factors are most similar, and how they together affect health outcomes. Clustering variables reduces the complexity of the dataset and makes parsing and understanding easier. Understanding these communities can help develop targeted interventions, for example, if specific health and demographic variables are clustered together, policies can be formulated to focus on addressing these specific factors. The insights obtained through group analysis can showcase various aspects of the determinants of health for mobile populations to decision-makers, thereby aiding in the development of more effective health strategies.

#### 3.2.4 Random forest model

The random forest algorithm is the first decision tree based model proposed by Breman, which predicts through statistical or machine learning algorithms. This model is known for its clear construction, clear explain ability, and high stabilization. The stochastic forest algorithm is made up of multiple decision trees, each of which randomly extracts sample information and generates multiple training sets. Each decision tree in the training set is a basic classifier, and the final predicted value is determined by the preponderance voting results of multiple trees. This algorithm is suitable for sort, regression, and prognosis work. Compared with a single decision tree, the random forest model performs better in sort and prognosis, and is less inclined to over matching. Select the best variable as the classification node in the decision tree and sort it according to its importance.

This paper chose the random forest algorithm to predict the priority order that affects the fitness status of migrant and regional populations. The main reason is that random forest introduces random selection attributes in the algorithm, recursively processes based on setting classification and termination criteria, and performs multiple regressions on each predictor variable (40). Therefore, this algorithm works effectively in large-scale datasets, has a slow response to over matching, and is not sensitive to final test data. So, the prediction results are more reliable. It also demonstrates the excellent performance of ensemble methods in practical applications.

The decision tree algorithm is developed based on the ID3 algorithm proposed by Quinlan. In 1984, Breiman et al. introduced the Gini coefficient as a segmentation criterion into the CART algorithm, using binary crystal trees. However, due to the decision tree being a single prediction model that is highly sensitive to data, it is difficult to optimize and improve prediction accuracy. To address this issue, we have developed an algorithm that integrates decision trees. Bootstrap Aggregating is an alternative method based on bootstrap sampling, which extracts *N* samples from a training set with an S-sample capacity to form a new training set. After *n* repetitions, generate *n* independent trace samples. The probability of not selecting each sample during the sampling process is calculated as (1−1/*N*) ^ *N*. The closer *N* approaches infinity as seen in Equation (13):


(13) $$\lim_{n\to \infty }{\left(1-\frac{1}{N}\right)}^{N}=e^{-1}\approx 0.3679$$

This means that when *N* is large enough, 36.79% of each guide sample will not be selected, but will become OOB (Out of Bag) data. The random forest algorithm is a ensemble method that uses a layout method to form a set of decision trees. It uses the Mean Increase in Accuracy (MDA) method to measure the priority score of attributes, thereby determining their importance in classification problems.

This study divided the sample into mobile population and regional population, and performed random forest prediction on each group to prioritize identifying factors that affect the reproductive behavior of mobile population and regional population. In order to ensure the randomness of the training sample set, the data was randomly sorted before model training. In order to ensure the consistency of the empirical results, a random seed output of 100,000 was set for the randomly generated seed player account system parameters (i.e., the size of the data sample is set to about 10 times, which maintains good performance than the consistent performance predicted by the model). The analysis results show that when the quantity of decision trees exceeds 500, the error rates of the movement of the random forest model and the fitness condition of the regional population tend to stabilize. The final dataset consists of half for training and the other half for testing. In order to obtain more accurate prediction results, we adjusted the number of repetitions for each segmentation and the number of random sampling variables.

The focus of this section is to choose the random forest algorithm to predict the priority order of factors that affect mobility and local population reproductive behavior. The main reason is that the random forest algorithm introduces random attribute selection, recursively sets classification and termination criteria, and performs multiple regressions on each predictor variable. This can effectively run on large-scale datasets, reduce the risk of overfitting, effectively respond to test data, and make prediction results more reliable. In practical applications, Random Forest demonstrates the excellent performance of ensemble algorithms. The priority prediction analysis based on random forest algorithm mainly consists of three stages: first, the number of repetitions is determined through OOB errors and validation errors. Secondly, the number of random sampling variables (Numvars) is determined based on the number of repetitions determined by OOB errors and validation errors. Finally, the priority score of the predicted variables is obtained through a random forest prediction model.

The machine learning models used, including PCA, BP neural network, cluster analysis, and random forest, all have inherent limitations. For example, PCA has interpretability, but if there are small changes in the data, it becomes unstable, leading to inconsistent results. BP neural network requires large computing resources, and if the dataset is small, it is easy to match it. Random forests alleviate this problem to some extent, but in highly imbalanced datasets, certain health outcomes may be underestimated. In addition, although machine learning algorithms are good at identifying data patterns, they do not naturally explain causal relationships. This boundary means that you must carefully interpret the results and imply that the discovered correlation may not directly represent causal relationships.

## 4 Results

### 4.1 Principal component analysis results

After data standardization, we conducted KMO (Kaiser Meyer Olkin) and Bartlett tests on 108,669 samples using SPSS software. This test is to confirm the suitability of the principal component analysis data for the 12 indicators used to establish an identity recognition index system (Table 1).

**Table 1:** KMO and Bartlett’s test.

Test		Value
KMO measure of sampling adequacy		0.688
Bartlett’s test of sphericity	Approx. chi-square	6447.378
	df	66
	Sig.	0.000

The test results show that the statistical value of KMO data is 0.688. In addition, the Kelvin squared value of Bartlett’s spherical calibration exceeds the critical value, with an attention level (Sig.) of 0.000, which is 0.05 lower than the traditional critical value. This indicates that it can refute the regression theory that the correlation matrix is not an identity matrix, and the data is suitable for analyzing factors. Therefore, these data are considered suitable for conducting principal component analysis.

The focus of subsequent analysis is the ratio of raw information captured by each major component. The first 12 main components with intrinsic values greater than 1 account for 73.641% of the original information. Based on past experience in analyzing the main components of health questionnaires, this ratio can fully reflect the information of all indicators. The three main components extracted can be divided into health factors, socio-economic factors, and demographic factors. These three main components integrate various aspects of the fitness condition of the floating population. The corresponding relationships between the main components and corresponding levels are detailed in Table 2.

**Table 2:** Weight of each principal component of health condition.

Principal Component	Main indicators	Label	Weight
Health factors	A01, A11, A12	F1	45.61
Socioeconomic factors	A06, A08, A9, A10	F2	30.19
Demographic factors	A2, A3, A4, A5, A7	F3	24.20

When constructing the identity recognition index, it is necessary to assign weight values to the three main components in order to comprehensively integrate the health status indicators of the floating population. The weight values of each major component are based on the ratio of dispersed contribution to cumulative dispersed contribution. By calculating the specific weight values of these 12 constituent elements (see Table 3), this study integrated the health status indicators of the floating population. The technical statistical data of the composite variables are shown in Table 3.

**Table 3:** Variable description statistics.

Variable	Mean	Standard deviation	Minimum	Maximum
Health	2.0439	0.9050	1.0000	3.0000
Health insurance	1.6412	0.3812	1.0000	2.0000
Gender	1.1067	0.2909	1.0000	2.0000
Age	2.0042	0.5396	1.0000	4.0000
Education level	3.6505	2.2864	1.0000	5.0000
Marriage	0.9256	0.4624	1.0000	2.0000
Work	1.3064	0.4051	1.0000	2.0000
Household Registration	1.1932	0.8403	1.0000	2.0000
Mobility range	2.1531	0.5513	1.0000	3.0000
Reasons for Mobility	1.9234	0.3691	1.0000	2.0000
Income	3.2651	0.9369	1.0000	5.0000
Health Education	1.6423	0.5416	1.0000	2.0000
Health record	1.2381	0.2543	1.0000	2.0000

The above table displays descriptive statistical data for the variables considered in principal component analysis. The mean and standard deviation of each variable reflect significant differences between factors such as health and income, which is crucial for understanding the health status of the floating population. For example, the average value of the “health” variable is 2.0439, the standard deviation is 0.9050, the minimum value is 1.000, and the maximum value is 3.000, which reflects significant differences in the health status of respondents in different populations. Similarly, the average value of the “income” variable is 3.2651, with a standard deviation of 0.9369, a minimum value of 1.0000, and a maximum value of 5.0000, indicating significant differences in the income levels of the floating population. In addition, in terms of health insurance, the average value is 1.6412 and the standard deviation is 0.3812, reflecting the differences in the coverage of health insurance for the floating population. This indicates that a large portion of the floating population does not have medical insurance, which may have adverse effects on their health. In addition, the average education level is 3.6505 with a standard deviation of 2.2864, which also reflects significant differences in the education level of the floating population, which may affect their ability to access knowledge and healthcare services (41).

This range of numbers and significant variability represent the diversity of population characteristics captured by the data. This makes principal component analysis particularly suitable as it can identify the factors that have the greatest impact on the health status of the floating population. By analyzing these variables, it is possible to determine which factors have the greatest impact on the health of the floating population, thereby providing a solid foundation for targeted intervention measures and policies for the health situation of the floating population (42). In short, the statistical description of these variables provides rich information, which helps us better understand and analyze the health status and influencing factors of the floating population, and provides scientific basis for relevant departments or policy makers to implement effective intervention measures, thereby improving the health status of China’s floating population.

### 4.2 BP neural network analysis results

A neural network is a topology structure consisting of three layers: input layer, hidden layer, and output layer, where each circle represents a neuron. Information flows from the input layer to the hidden layer, and then to the output layer. This study divides the collected floating population samples into a test set and a training set, and predicts the health status of the floating population through BP neural network.

**Figure 2:**
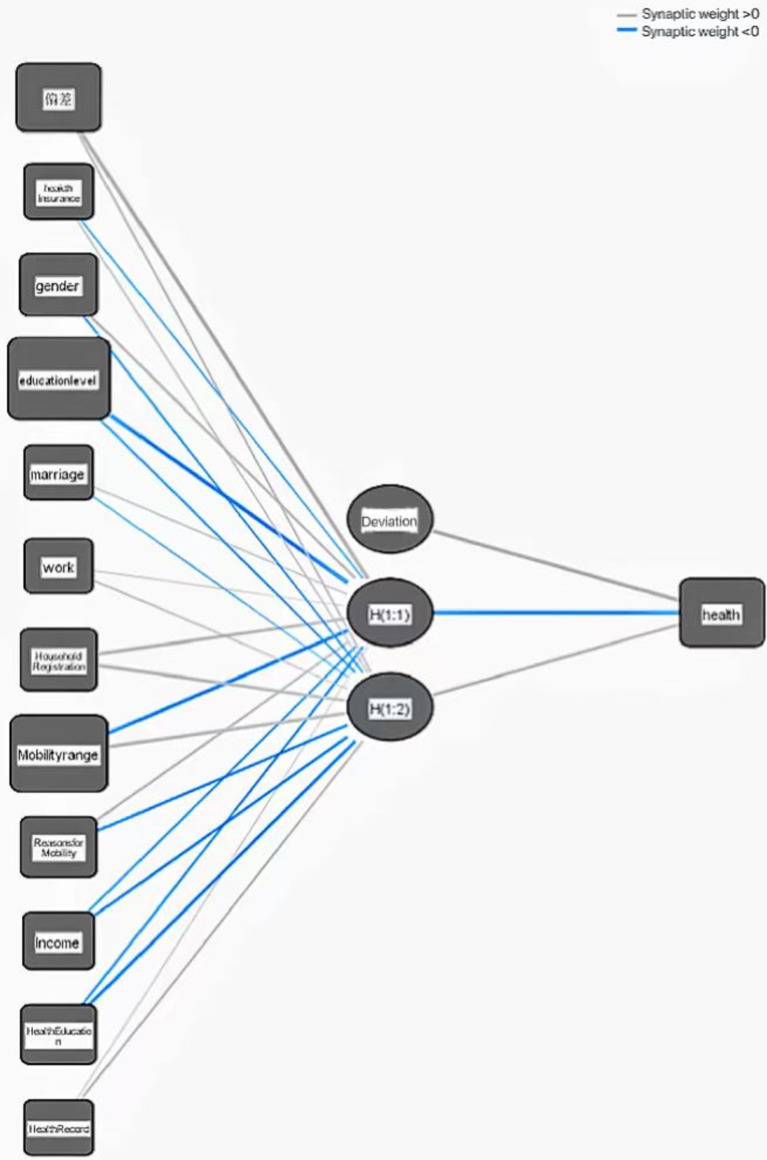
Health model of floating population based on BP neural network.

After multiple experiments, a three-layer BP neural network with multiple inputs and a single output function was ultimately selected as the national unemployment risk warning model for the floating population. The model consists of 12 input layer neurons, 2 hidden layer neurons, and 1 output layer neuron, as shown in Figure 2. In order to avoid the impact of manual sample selection on the training results, a random sampling method was used to optimize the training results. The final model selected 7 samples as training samples and 2 samples as testing samples. In the test set, the relative error of the model was 3.6%, demonstrating the high accuracy of the simulation results. Meanwhile, synaptic weights are key components in neural networks, representing the strength of connections between neurons. By adjusting these weights, neural networks can learn and adapt to changes in the external environment. In the above figure, synapses with weights greater than 0 are marked in gray, while those with weights greater than 0 are marked in blue.

**Figure 3:**
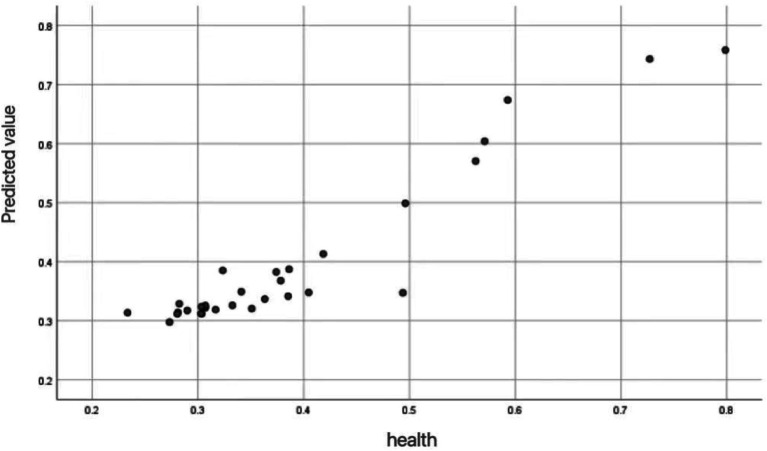
Scatter plot for health prediction of mobile population.

The effectiveness of neural network models in predicting the health status of mobile populations can be visualized through the calculation points in Figure 3. The displayed calculation chart shows the relationship between the fitness values of the floating population and the actual fitness values, using a neural network model. Each point in the chart represents an individual’s health prediction, the horizontal axis represents the actual health value, and the vertical axis represents the predicted health value.

From the Figure 3, it can be observed that there is a positive correlation between predicted values and actual health values. As the actual health value increases, the predicted value also tends to increase. This indicates that the neural network model can predict health status well, with most points concentrated on the diagonal. The model can accurately capture the potential relationship between health influencing factors and health outcomes. The distribution of these points indicates that the model’s predictions are very close to the actual values, which further supports the high average accuracy and low mean squared error in the research report.

**Figure 4:**
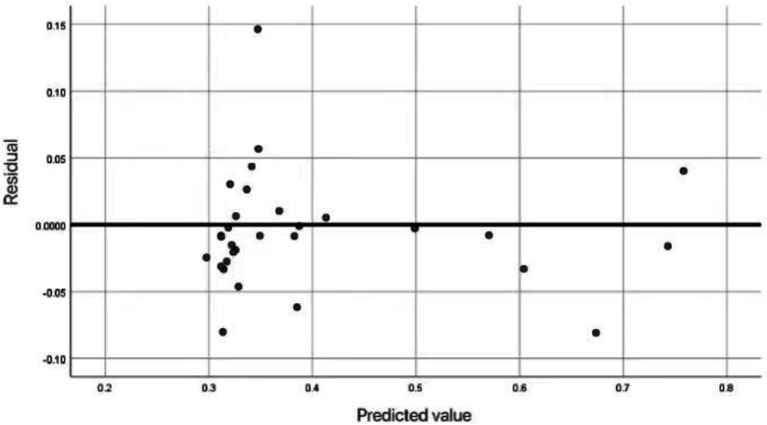
Scatter residual map of health prediction of floating population.

In the prediction of the fitness condition of the floating population, in order to further verify the correctness of the neural network model, the relationship between the predicted values and the actual residuals was described. Figure 4 shows the relationship between predicted health values and actual residuals for each respondent. By analyzing the residual distribution, the predictive performance and error characteristics of the model can be evaluated.

The residual chart shown in Figure 4 illustrates the difference between the predicted health values (x-axis) and the actual residuals (y-axis) of the respondents in the neural network model. Each point represents the respondent, and the margin is calculated based on the difference between the observed and predicted values. The horizontal line represents the perfect accuracy of the prediction. The dense residuals around this line mean that the neural network model has high prediction accuracy and minimizes the deviation between predicted values and actual health outcomes. This further confirms the reliability of the model’s health status prediction.

In order to evaluate the influence of various factors on the fitness status of the floating population, a neural network model was adopted. This model evaluates the importance of various predictive factors in various regression models (linear, logical, square, logarithmic) and the importance of normalization. The results are as follows:

The results in Table 4 emphasize the varying degrees of importance of different independent variables in predicting the health outcomes of the floating population, which are determined by their standardized significance values. The most influential factor is health insurance, with a standardized significance of 70.0%. This indicates that medical insurance plays a crucial role in predicting health outcomes, emphasizing the importance of obtaining medical insurance in maintaining and improving the health of migrant populations. Health education (68.8%) and health records (64.1%) also play an important role, indicating that a better understanding of healthcare knowledge among migrant populations can encourage them to make healthier choices and seek necessary medical measures. Comprehensive and accessible records of their health status will have a significant impact on the health of migrant populations, improving their own health conditions. Interestingly, among the predictive factors, gender (18.1%) has the lowest importance, while other factors have a greater direct impact on health status. Among other variables, such as education level (43.2%), registered residence (51.5%), reasons for migration (60.2%) and marital status (42.2%), although they have different degrees of prediction and impact on the health of migrant population, they also play a certain role. Because the level of education often affects the various channels and willingness of migrant populations to obtain health knowledge and information. At the same time, registered residence will also have a certain impact on the access to regional health resources. The reasons for migration may have different impacts on health due to different employment or educational opportunities. In addition, the marital status of the floating population also has a certain impact on the social support system and mental health.

**Table 4:** Importance of independent variables.

Independent variable	Model	Sig.	Normalized sig.
Gender	Linear	0.024	18.1%
Health record	Linear	0.096	64.1%
Health insurance	Logistic	0.114	70.0%
Income	Linear	0.105	62.9%
Educational level	Power	0.095	43.2%
Household registration	Logistic	0.080	51.5%
Mobility range	Linear	0.130	53.0%
Reasons for mobility	Power	0.091	60.2%
Marriage	Linear	0.068	42.2%
Health education	Logarithmic	0.109	68.8%
Work	Linear	0.088	58.0%

Overall, the above analysis emphasizes the multifaceted factors that affect the health of migrant populations, which can help policy makers implement targeted interventions and policies, guiding decision-makers to focus on improving the most important areas of health outcomes.

Due to the cyclical changes in economic development affecting every determining factor, the model is adjusted based on the economic cycle reflecting existing data. As seen in Equations (14–17).

The linear prediction model is as follows:


(14) $$y=\beta_{0}+\beta_{1}x_{i}+\mu_{i}$$

The logarithmic curve prediction model is as follows:


(15) $$y=\beta_{0}+\beta_{1}\ln\left(x_{i}\right)+\mu_{i}$$

The logistic curve prediction model is as follows:


(16) y=11ξ+β0β1x+μi

The power function prediction model is as follows:


(17) $$y=\beta_{0}\left(x^{\beta_{1}}\right)+\mu_{i}$$

Using survey data, we predicted the determinants of health status for the migrant population. The prediction results are denoted as yi, representing the predicted value of the i determinant (specific prediction results are omitted due to space constraints). Subsequently, based on the actual values xi and the predicted values yi of each determinant, we calculated the accuracy and the mean square error (formulas provided below). As seen in Equation (18).


(18) $$MSE=\frac{1}{n}\sum {\left(x_{i}-y_{i}\right)}^{2}$$

The average accuracy was 97.8%, with a maximum value of 100% and a minimum value of 96.5%. The mean square error was 0.0018%, indicating that the prediction results were highly accurate.

### 4.3 Cluster analysis results

In the context of predicting the health status of pure mobile populations, cluster analysis can provide deep insights into the relationship between independent variables and clustering. By identifying clusters of variables with similar characteristics, we can better understand the potential patterns and correlations that affect health outcomes. Here is a cluster analysis of the variables used in neural network models, determining grouping methods, and explaining their impact on health status prediction.

**Figure 5:**
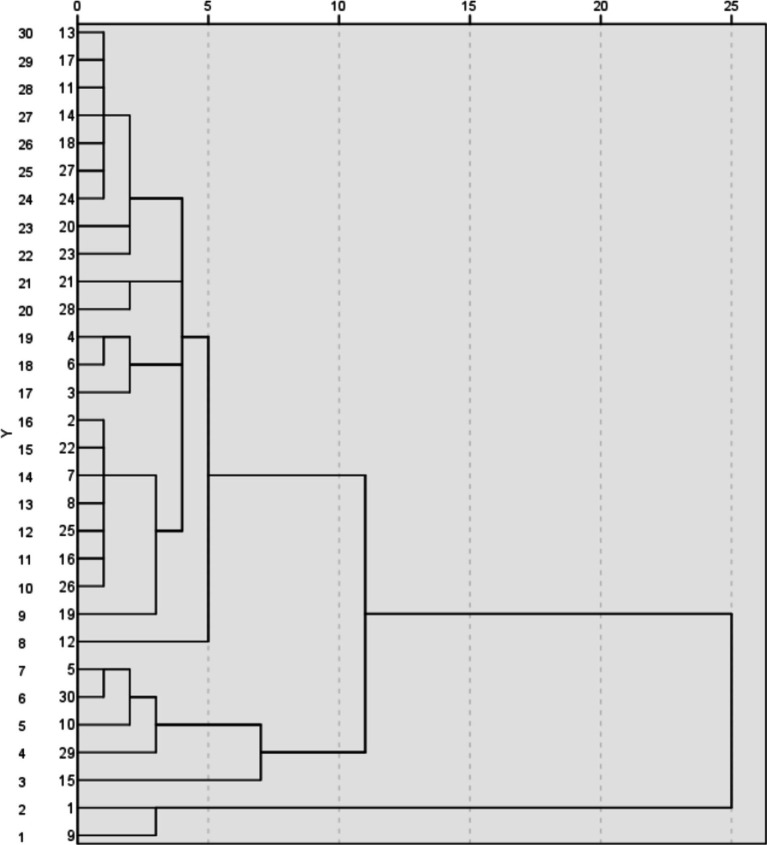
Average link (intergroup) pedigree chart.

Figure 5 is a cluster hierarchical diagram showing the relationships and intrinsic similarities between different variables that affect the health status of the migrant population. This image shows two main clusters with a degree of difference of approximately 20, which is a key issue in the analysis. Cluster 1: Group variables such as health (1), health insurance (2), gender (9), age (19), etc. together. Cluster 2: Form clear groups with different variables such as education level (3), marriage (4), occupation (7), and income (14). In each major cluster, further segmentation represents more specific variable grouping. Subgroup 1.1: Includes variables related to direct health indicators, such as health (1) and health insurance (2). Subgroup 1.2: Contains demographic variables such as gender (9) and age (19). Subgroup 2.1: Includes variables related to socio-economic factors, such as education level (3) and income (14). Subgroup 2.2: Includes variables related to social status and employment, such as marriage (4) and occupation (7). The height of variable connections indicates their level of non-similarity. Lower connection points indicate higher similarity between variables. At a higher level of non-similarity, the segmentation of the first major cluster shows that the variables in each major cluster are more similar than those in other clusters.

Through hierarchical cluster analysis, the tree chart shows that the variables that affect the fitness condition of the floating population are mainly divided into two categories: health and demographic factors. Further subdivisions within each main cluster represent more specific sets of variables. Cluster 1 (Health and Demographic Factors): This cluster contains direct health indicators and demographic variables, indicating a close correlation between health status and basic demographic factors. Cluster 2 (Socio economic factors): This cluster contains socio-economic variables such as education, income, marital status, and employment, which have a significant impact on health outcomes. By identifying these clusters, we can better understand the multifaceted nature of health determinants for mobile populations. These insights have played an important role in targeted interventions and policy development to improve the health status of these populations.

In summary, the hierarchical clustering analysis indicates that the variables affecting the health status of the floating population can be roughly divided into demographic factors related to health and socio-economic factors. This detailed understanding helps to develop more evidence-based and effective interventions and policies to promote the health and well-being of migrant populations (43). When addressing the health issues of the floating population, stakeholders can focus on each of the most important determining factors and develop more targeted strategies to comprehensively improve the health status of China’s floating population.

### 4.4 Random forest model results

According to the principle of the random forest algorithm mentioned above, due to a sufficiently large number of repetitions, it is possible to converge the out of bag error and validation error. Therefore, when adjusting the number of random sampling variables, it can be confirmed that these errors are not caused by the number of repetitions but by the variables. In fact, in the training set containing 85,219 samples, the number of repetitions gradually increased from 100, and Figure 4 shows the relationship between the number of repetitions and the out of bag error and validation error. As shown in Figure 4, as the number of repetitions increases, the out of bag error gradually stabilizes, and the validation error shows a decreasing trend. Finally, when the number of repetitions reaches 500, the error of the random forest model on the reproductive behavior of the local population tends to stabilize. At this point, the out of bag error is maintained at around 38%, and the validation error is maintained at around 46%. Therefore, in the random forest model, selecting 500 integers as factors affecting local population reproductive behavior can achieve better predictive performance.

Therefore, for the mobile population, the seed count is set to 100,000, including a training set of 23,450 samples, gradually increasing the number of repetitions starting from 100. As shown in Figure 5, as the number of repetitions increases, the out of bag error and validation error will gradually stabilize. When the number of repetitions reaches 500, the out of bag error is maintained at around 32%, and the validation error is maintained at around 61%. Therefore, by fixing the number of repetitions to 500, the stability and reliability of the prediction results can be ensured, and better predictive performance can be achieved.

Based on the above research, the number of repetitions and the number of random sampling variables of the random forest prediction model were determined. After setting up the prediction model based on this, the ranking and comparison results of the influencing factors of mobile phones and regional population health status are detailed in Table 5.

**Table 5:** Comparison of the priority ranking of factors affecting the health status of floating population and local population.

	Floating population	Local population
1	Health insurance	Health record
2	Health education	Health insurance
3	Income	Work
4	Health record	Mobility range
5	Reasons for mobility	Health education
6	Work	Marriage
7	Education level	Income
8	Mobility range	Education level
9	Age	Reasons for mobility
10	Household registration	Age
11	Marriage	Household registration
12	Gender	Gender

Table 5 shows a comparison of the priorities of the migrant population and factors affecting the fitness condition of the local population. For the floating population, health insurance, health education and income are the top three factors. This indicates that access to health services and health information is often hindered, and therefore health services and health-related knowledge are very important for migrant populations (44). Income also plays an important role because it affects your ability to pay for medical care and maintain a healthy lifestyle.

In contrast, for the regional population, health records, health insurance and jobs are the three most important factors (45). This shows that maintaining comprehensive health records and health insurance is very important for the health management of the regional population. In addition, the stability of employment and working conditions also have a strong impact on their health. Health records are the most important factor in the regional population, emphasizing the importance of continuous health monitoring and regular health checkups. More accessible than the migrant population.

In summary, the comparison of priorities in Table 6 highlights the differences in determinants of health between the migrant and local populations. For both groups, health insurance and health education are important, but of different relative importance. The analysis emphasizes the need to develop health policies and interventions tailored to the specific needs of each group. For the migrant population, improving access to health insurance and education is important, and for the local population, maintaining a comprehensive health record and stable employment is key (46).

**Table 6:** Comparison of the priority ranking of factors affecting the health status of floating population and local population in subregions.

Ranking	Eastern cities		Central cities		Western city	
	Floating population	Local population	Floating population	Local population	Floating population	Local population
1	Income	Health insurance	Health insurance	Income	Income	Health insurance
2	Health insurance	Income	Income	Health insurance	Health insurance	Income
3	Work	Health education	Health education	Work	Work	Health record
4	Health education	Education level	Work	Education level	Health education	Mobility range
5	Education level	Work	Education level	Health education	Education level	Health education
6	Reasons for mobility	Age	Reasons for mobility	Reasons for mobility	Reasons for mobility	Work
7	Age	Reasons for mobility	Mobility range	Age	Marriage	Reasons for mobility
8	Mobility range	Marriage	Age	Health record	Mobility range	Marriage
9	Marriage	Mobility range	Marriage	Mobility range	Age	Education level
10	Health record	Health record	Health record	Marriage	Health record	Age
11	Household registration	Household registration	Household registration	Household registration	Gender	Household registration
12	Gender	Gender	Gender	Gender	Household registration	Gender

The priority of elements affecting the well-being status of floating population and local population in this study is to further study the health status of floating population and local population in eastern, central and western cities of each region of the sample, and the priority order of influencing factors is analyzed. To investigate the factors influencing fertility behavior of regional populations, we built a random list model containing 100,000 seeds and used a training set containing 23,852 population samples from eastern urban areas. Figure 5 shows the relationship between self-burden error, validation error, and number of repetitions of the population in the eastern urban area. As shown in Figure 5, when the number of repetitions reaches 500, the out-of-pocket error is about 0.43, and the verification error is 0.48, which is very stable. Therefore, the number of repeated times is fixed to 500, which can ensure the stability and reliability of the prediction results and improve the prediction performance. In the case of central cities, after setting up a random forest model with 100,000 seeds in order to determine the influencing factors on health status, the training set included 23,611 floating population samples and 25,396 regional population samples. When the number of repetitions reaches 500, the out-of-pocket error for the entire movement is about 0.13, the in-pocket error for the entire region is 0.44, and the verification error is 0.68 and 0.49, respectively. Therefore, the number of repeated times is fixed to 500, which can ensure the stability and reliability of the prediction results and improve the prediction performance. In the case of western cities, in order to determine the influencing factors of health status, a random forest model of 100,000 seeds was established, and the training set included 9,624 floating population samples and 12,813 regional population samples. When the number of repetitions reaches 500, the out-of-pocket error for the entire movement is about 0.37, the in-pocket error for the entire region is 0.41, and the validation error stabilizes at 0.56 and 0.46, respectively. Therefore, the number of repeated times is fixed to 500, which can ensure the stability and reliability of the prediction results and improve the prediction performance.

Table 6 shows the priorities affecting the health status of urban migrants and regional populations in the east, central and west. The analysis highlights regional differences and commonalities in the determinants of health across regional population groups.

In eastern cities, the main factors determining the fitness of the migrant population are revenue and medical insurance, which reflects the central role of medical care in stabilizing the economy and maintaining health. For the regional population, health insurance and income are the most important factors, illustrating the similarity of the two health needs in the region. But work and health education is more important for the migrant population (47), which reflects the importance of employment and health awareness for more migrant groups. The impact of education level and age on the local population is even more pronounced, highlighting the impact of educational outcomes and age-related health problems. In central cities, medical insurance and income are again the main factors for migrants, which is consistent with the pattern in eastern cities. But health education and jobs are also high on the list, highlighting the need for health literacy and jobs. For the local population, income and health insurance are equally important, but education level and employment are the most important factors, reflecting the impact of financial, education and employment factors on health (48). Factors related to mobility, such as the cause and extent of mobility, are relatively important for both populations, indicating the impact of mobility on the health of central cities. In Western cities, migrants prioritize income, health insurance, and work, highlighting the fundamental role of economic and employment factors in health outcomes. Health insurance and income remain important to the regional population, but health records and mobility have also become central factors reflecting the importance of troop and regional mobility. The level of education and health education is necessary for the mobile population, and the regional population is based on the stability and multiple health effects of the life stage, emphasizing the importance of age and marriage (16).

According to the analysis, income and health insurance are consistently the main factors for both migrant and local populations in all regions, highlighting the universal importance of financial security and health care. But the importance of factors such as jobs, health education, and mobility varies by region and demographic group. For example, working in eastern and central cities is more important for migrants, while range of movement is more important for regional populations in western cities. In addition, unique factors such as mobile population and age have different importance in different regions, indicating that populations in different geographic areas face different challenges and health determinants (49). Overall, the analysis in Table 6 reveals commonalities and regional variability in elements influencing the fitness status of floating and local populations. These perspectives can be used as a reference to develop health policies and interventions tailored to the specific requirements and circumstances of different regions, which can ultimately improve health outcomes for all population groups.

## 5 Discussion

BP neural network is a widely used model in machine learning to analyze the fitness affecting elements of floating population. The model, which is based on multiple input variables such as income, education level, work environment and access to health care, shows high accuracy in predicting health outcomes. The BP neural network identified income and education level as the most important predictors of health status, which is consistent with existing literature emphasizing the importance of socioeconomic factors. The nonlinear nature of BP neural networks can capture complex interactions between variables, providing detailed insights into how different factors work together to affect health.

Cluster analysis can group the floating population according to the similarity of health characteristics and influencing factors. The analysis reveals different clusters, each with its own characteristics. For example, one group consists primarily of young, low-income individuals with limited access to medical services and poor health. Another group includes the steadily employed middle-aged, who enjoy better health although they have access to appropriate medical services. This fragmentation highlights heterogeneity within migrant populations, suggesting that health interventions should address the unique needs of different sub algae rather than one size fits all.

Due to its stability and ability to handle large datasets and high-level data, the random forest model is used to rank various health impact factors. The model classifies health insurance, health education and income as the top 3 factors affecting the well-being condition of the floating population. Fitness insurance was identified as the most critical factor, and the importance of increasing the migrant population’s access to medical services was highlighted. Health education has also been identified as a core element that can substantially improve health outcomes if health-related information and education are provided. The importance of income validates the BP neural network findings, underscoring the role of economic stability in health.

Compare the results of BP neural networks, cluster analysis, and random cell models to reveal commonalities and unique insights between them. All three methods emphasize the importance of income in determining socioeconomic factors, particularly health status. But random list models uniquely emphasize the central role of health insurance and health education, and BP neural networks provide a detailed understanding of the interactions of complex variables. The Group’s analysis presents multiple characteristics of the migrant population and highlights the need for targeted interventions.

These findings have important practical implications for policy makers and medical providers to improve the health status of migrant populations. First, the findings highlight the importance of strengthening health education programs. By investing in comprehensive health education, policymakers can equip migrant populations with the knowledge and technology necessary to take healthier actions and make informed health decisions (50). Secondly, it is important to promote access to fitness insurance and medical services. Policy makers should strive to reduce barriers to access to medical services, so that the migrant population can have timely and adequate access to medical services. Healthcare providers can use this information to develop interventions to address the specific health needs of migrant populations, such as mobile clinics or telemedicine services. By integrating these strategies, policymakers and healthcare providers can create more supportive environments for migrants to achieve better health outcomes (51).

The findings of this study make a significant contribution to the broad theoretical framework of factors determining the health of migrant populations. Traditional theories, such as the Social Determinants of Health (SDH) framework, emphasize the importance of socioeconomic, environmental, and behavioral factors in health outcomes. Our findings are consistent with these theories, suggesting that factors such as employment status, housing conditions, and access to medical services are key determinants of the health of China’s migrant population (52). In addition, the application of machine learning algorithms can identify and analyze complex non-linear relationships between these determinants and health outcomes, leading to a more detailed understanding of how these factors interact. As such, this study combines advanced analytical methods, provides hidden models, extends the SDH framework, and provides empirical evidence to guide public intervention. Placing our findings in this established theoretical context, we highlight the relevance and applicability of our research to national and international health policy discussions.

All in all, the factors affecting the health of floating population can be fully understood by using BP neural network, cluster analysis and random sampling model. The consistency of this approach strengthens the evidence base for prioritizing health insurance, education, and socio-economic assistance, and points the way for policy interventions. Future research is needed to continue to refine these models and explore other factors to develop more targeted and effective health strategies for migrant populations.

## 6 Conclusion

In summary, the study uses advanced machine learning techniques to analyze the health elements of the migrant and local populations and determine priorities. Using random lists, BP neural networks, and cluster analysis, we developed a comprehensive understanding of the unique and common health influences of these individuals. Based on stability and interpretability, random forest models effectively deal with the complexity and variability of health data, providing reliable predictions and valuable variable importance levels. Although BP neural networks are computationally demanding and so can be suitable, they have shown a strong ability in modeling nonlinear relationships in data, cluster analysis is meaningful, and identifying subgroups and differences in our understanding of people’s health patterns helps to strengthen.

The findings highlight the importance of elements such as health-insurance, health education, and income for migrants, with health records, employment, and mobility required for regional populations (53). These results highlight the need to tailor health policies and interventions to the specific needs of each group.

The results of this study have important practical implications for designing targeted interventions to improve the fitness status of the migrant population. Grounded on BP neural network, cluster analysis and random sampling model, one of the main applications is to establish a comprehensive health insurance plan for the floating population (54). Policy makers can expand access to health care and work to provide migrants with access to quality health care that is affordable anywhere. In this way, portable medical insurance plans that are not linked to a specific region can be developed, solving the liquidity problem faced by the group (55). In addition, governments and non-governmental organizations can work together to provide subsidies or financial incentives to encourage migrants to participate in these health insurance programs.

Another important application of this research is the implementation of health education and promotion programs for the floating population. The study highlights the importance of health education in improving health outcomes. Therefore, targeted health education programs can be designed to address the specific needs and challenges of the migrant population. These projects could focus on preventive care, healthy lifestyle choices and raising awareness about the effective use of available health services. Mobile health groups and digital health platforms can be used to engage with mobile users and access relevant health information in a timely manner. In addition, community health initiatives supported by local governments and NGOs can provide ongoing support and resources for migrants to foster healthier, more informed communities (56). By incorporating these applications into the framework of health policies, great progress can be made in improving the overall health status of the migrant population.

In order to emphasize the practical significance of our findings, this study analyzed the successful intervention cases in depth. For example, Wang et al. (57) found that targeted health education programs greatly improved health literacy and outcomes among immigrant populations. Similarly, research by Li et al. has shown that the implementation of mobile clinics can improve health status by increasing access to essential health services in underserved communities. These examples highlight the effectiveness of our recommendations in practical applications and provide specific guidance for policymakers and staff. By integrating these proven strategies, our research provides actionable insights for improving public health services and health education for immigrant populations, and ultimately promoting better health outcomes.

## Data Availability

The original contributions presented in the study are included in the article/supplementary material, further inquiries can be directed to the corresponding author.

## References

[1] Kwon K Pan J Guo Y Ren Q Yang Z Tao J. Demirjian method and Willems method to study the dental age of adolescents in Shanghai before and after 10 years. Folia Morphol (Warsz). (2023) 82:346–58. doi: 10.5603/FM.a2022.0025, PMID: 35285510

[2] Chang J Deng Q Hu P Guo M Lu F Su Y. Geographic variation in mortality of acute myocardial infarction and association with health care accessibility in Beijing, 2007 to 2018. J Am Heart Assoc. (2023) 12:e029769. doi: 10.1161/JAHA.123.029769, PMID: 37301748 PMC10356049

[3] Kan X Zhu S Zhang Y Qian C. A lightweight human fall detection network. Sensors. (2023) 23:9069. doi: 10.3390/s23229069, PMID: 38005456 PMC10674212

[4] Sharareh N Zheutlin AR Qato DM Guadamuz J Bress A Vos RO. Access to community pharmacies based on drive time and by rurality across the contiguous United States. J Am Pharm Assoc. (2024) 64:476–82. doi: 10.1016/j.japh.2024.01.004, PMID: 38215823

[5] Rzodkiewicz LD Turcotte MM. Two duckweed species exhibit variable tolerance to microcystin-Lr exposure across genotypic lineages. Harmful Algae. (2024) 131:102548. doi: 10.1016/j.hal.2023.102548, PMID: 38212081

[6] Fan C Li S Liu Y Jin C Zhou L Gu Y. Using social media text data to analyze the characteristics and influencing factors of daily urban green space usage-a case study of Xiamen, China. Forests. (2023) 14:1569. doi: 10.3390/f14081569

[7] Chen L Zeng H Wu L Tian Q Zhang N He R. Spatial accessibility evaluation and location optimization of primary healthcare in China: a case study of Shenzhen. Geohealth. (2023) 7:e2022GH000753. doi: 10.1029/2022GH000753, PMID: 37200630 PMC10187614

[8] Liu W Chen R. Migration networks pattern of China's floating population from the perspective of complex network. Chin Geogr Sci. (2024) 34:327–41. doi: 10.1007/s11769-023-1402-9

[9] Chen J Li H Luo S Xie J Su D Kinoshita T. Rethinking urban park accessibility in the context of demographic change: a population structure perspective. Urban For Urban Green. (2024) 96:128334. doi: 10.1016/j.ufug.2024.128334

[10] Wei Z Xie R Tang Q Chan EHW Chen Y Xiao L. Planning strategies for promoting spatial accessibility of healthcare facilities in shrinking cities: a case study of Lufeng in China. J Urban Plan Dev. (2024) 150:05024002. doi: 10.1061/JUPDDM.UPENG-4696

[11] He Y Xu S Fu T Zhao DN. The impact of China's family floating population on the participation of medical Insurance in the Inflow Areas. J Multidiscip Healthc. (2024) 17:949–57. doi: 10.2147/JMDH.S451303, PMID: 38465326 PMC10921892

[12] Huan L Shi M Wang X Gu W Zhang B Liu X. Morphological characteristics and genetic diversity of floating and attached Ulva prolifera--a case study in the Yellow Sea, China. Mar Pollut Bull. (2023) 195:115468. doi: 10.1016/j.marpolbul.2023.115468, PMID: 37666140

[13] Chong F Spencer M Maximenko N Hafner J McWhirter AC Helm RR. High concentrations of floating neustonic life in the plastic-rich North Pacific garbage patch. PLoS Biol. (2023) 21:e3001646. doi: 10.1371/journal.pbio.3001646, PMID: 37141195 PMC10159152

[14] Fei C Zhu Y Jiang L Zhou H Yu H. Social integration, physical and mental health and subjective well-being in the floating population-a moderated mediation analysis. Front Public Health. (2023) 11:1167537. doi: 10.3389/fpubh.2023.1167537, PMID: 37483925 PMC10356978

[15] Jiang J Zhou Y Xu J Wang Z. The vulnerability of international floating populations to sexually transmitted infections: a qualitative study. Healthcare. (2023) 11:1744. doi: 10.3390/healthcare11121744, PMID: 37372862 PMC10298312

[16] Hu Y Wu X Zhang Z. Socioeconomic status and self-rated health among Chinese rural-to-urban migrants: evidence from the 2018 China migrants dynamic survey. Health Place. (2022) 73:10273434954539

[17] Li X Zhang W Liu Y. Work stress, social support, and mental health of migrant workers in China. Int J Environ Res Public Health. (2020) 17:1261.32075326

[18] Alam MS Tabassum NJ Tokey AI. Evaluation of accessibility and equity to hospitals by public transport: evidence from six largest cities of Ohio. BMC Health Serv Res. (2023) 23:598. doi: 10.1186/s12913-023-09588-0, PMID: 37291565 PMC10251528

[19] Jorg R Haldimann L. Mhv3sfca: a new measure to capture the spatial accessibility of health care systems. Health Place. (2023) 79:102974. doi: 10.1016/j.healthplace.2023.102974, PMID: 36708664

[20] Bhuiyan MA Liu Z Meng F. Measurement and difference analysis of multidimensional poverty of floating population. Kybernetes. (2024) 53:1168–80. doi: 10.1108/K-07-2022-0943

[21] Wang C Leitner M Paulus G. Multiscale analysis of spatial accessibility to acute hospitals in Carinthia, Austria. ISPRS Int J Geo Inf. (2023) 12:491. doi: 10.3390/ijgi12120491

[22] Wang X He J Liao TF Gu G. Does air pollution influence the settlement intention of the floating population in China? Individual heterogeneity and City characteristics. Sustainability. (2023) 15:2995. doi: 10.3390/su15042995

[23] Liang Y Lu P. The effect of health on the labor supply of elderly migrants in China. Soc Sci Med. (2020) 255:112991

[24] Zhou H Yang X Liu Z. Social support and mental health among migrant workers in urban China: a cross-sectional study. BMC Public Health. (2021) 21:1–9.33388037

[25] Lin X Mao X Ai F Yao W. Factors influencing utilization of communicable disease prevention and treatment education among the floating population: a cross-sectional study in China. BMC Public Health. (2023) 23:207. doi: 10.1186/s12889-023-15126-8, PMID: 36721260 PMC9887564

[26] Charlesworth CJJ Nagy D Drake C Manibusan B Zhu JM. Rural and frontier access to mental health prescribers and nonprescribers: a geospatial analysis in Oregon Medicaid. J Rural Health. (2024) 40:16–25. doi: 10.1111/jrh.12761, PMID: 37088967 PMC10590824

[27] Liang Y Xie Z Chen S Xu Y Xin Z Yang S. Spatial accessibility of urban emergency shelters based on Ga2sfca and its improved method: a case study of Kunming, China. J Urban Plan Dev. (2023) 149:05023013. doi: 10.1061/JUPDDM.UPENG-4325

[28] Chawla H Singh SK Haritash AK. Reversing the damage: ecological restoration of polluted water bodies affected by pollutants due to anthropogenic activities. Environ Sci Pollut Res. (2023) 31:127–43. doi: 10.1007/s11356-023-31295-w, PMID: 38044406

[29] Wei Z Bai J. Measuring spatial accessibility of residents' medical treatment under hierarchical diagnosis and treatment system: multi-scenario simulation in China. PLoS One. (2023) 18:e0282713. doi: 10.1371/journal.pone.0282713 37036836 PMC10085051

[30] Parthiban A Sathish S Suthan R Sathish T Rajasimman M Vijayan V. Modelling and optimization of thermophilic anaerobic digestion using biowaste. Environ Res. (2023) 220:115075. doi: 10.1016/j.envres.2022.115075, PMID: 36566967

[31] Wang F Zeng Y Liu L Onega T. Disparities in spatial accessibility of primary care in Louisiana: from physical to virtual accessibility. Front Public Health. (2023) 11:1154574. doi: 10.3389/fpubh.2023.1154574 37143988 PMC10151773

[32] Sun X Cheng Y Tao Z. Spatial accessibility and equity of residential care facilities in Beijing from 2010 to 2020. Health Place. (2024) 86:103219. doi: 10.1016/j.healthplace.2024.103219 38467103

[33] Chen X Tan X Li J. The impact of working conditions and lifestyle on health among internal migrants in China: a systematic review. J Public Health. (2021) 43:e216–23.

[34] Huang J Chen Y Liu G Tu W Bergquist R Ward MP. Optimizing allocation of colorectal cancer screening hospitals in Shanghai: a geospatial analysis. Geospat Health. (2023) 18. doi: 10.4081/gh.2023.1152 37401409

[35] Guo X Zeng S Namaiti A Zeng J. Evaluation of supply-demand matching of public health resources based on Ga2sfca: a case study of the central urban area of Tianjin. ISPRS Int J Geo Inf. (2023) 12:156. doi: 10.3390/ijgi12040156

[36] Sarkar DJ Das Sarkar S Kumar VS Chanu TN Banerjee T Chakraborty L. Ameliorative effect of natural floating island as fish aggregating devices on heavy metals distribution in a freshwater wetland. Environ Pollut. (2023) 336:122428. doi: 10.1016/j.envpol.2023.122428, PMID: 37611791

[37] Ogawa M Mitani Y. Distribution and composition of floating marine debris in Shiretoko peninsula, Japan, using opportunistic sighting survey. Mar Pollut Bull. (2024) 201:116266. doi: 10.1016/j.marpolbul.2024.116266, PMID: 38522339

[38] Gao P Qi W Liu SH Liu Z Pan ZH. Moving to a healthier city? An analysis from China's internal population migration. Front Public Health. (2023) 11:1132908. doi: 10.3389/fpubh.2023.1132908 36860386 PMC9969190

[39] Hendrix N Warkaye S Tesfaye L Woldekidan MA Arja A Sato R. Estimated travel time and staffing constraints to accessing the Ethiopian health care system: a two-step floating catchment area analysis. J Glob Health. (2023) 13:04008. doi: 10.7189/jogh.13.04008, PMID: 36701563 PMC9880518

[40] Gao Y Kan X Cheng J Zheng S Chen M. Accessibility evaluation of a newly planned high-speed Railway Station in a metropolitan Core area based on a modified two-step floating catchment area method. J Urban Plan Dev. (2024) 150:05024012. doi: 10.1061/JUPDDM.UPENG-4306

[41] Xing Y Zhang L Zhang Y He R. Relationship between social interaction and health of the floating elderly population in China: an analysis based on interaction type, mode and frequency. BMC Geriatr. (2023) 23:662. doi: 10.1186/s12877-023-04386-z, PMID: 37845627 PMC10580520

[42] Chen L Chen T Lan T Chen C Pan J. The contributions of population distribution, healthcare resourcing, and transportation infrastructure to spatial accessibility of health care. Inquiry. (2023) 60:469580221146041. doi: 10.1177/00469580221146041, PMID: 36629371 PMC9837279

[43] Jin M Deng Q Wang S Wei L. Equity evaluation of elderly-care institutions based on Ga2sfca: the case study of Jinan, China. Sustainability. (2023) 15:16943. doi: 10.3390/su152416943

[44] Liu Y Xiao J Fan S Miao XX Yuan C Zang Y. Distribution and diversity of the sympatric macroalgae of the pelagic Sargassum horneri in the yellow and East China seas. Aquat Bot. (2023) 188:103683. doi: 10.1016/j.aquabot.2023.103683

[45] Silva JP Santos CJ Torres E Martínez-Manrique L Barros H Ribeiro AI. A double-edged sword: Residents' views on the health consequences of gentrification in Porto, Portugal. Soc Sci Med. (2023) 336:116259. doi: 10.1016/j.socscimed.2023.116259, PMID: 37806145

[46] Wang L Zhao Y Shi Y Liu H Li H Liu J. The interaction effects between exposure to ambient Pm2.5 and economic development on the settlement intention for floating population in China. Environ Sci Pollut Res. (2023) 30:67217–26. doi: 10.1007/s11356-023-27043-9, PMID: 37103706

[47] Soukhov A Páez A Higgins CD Mohamed M. Introducing spatial availability, a singly-constrained measure of competitive accessibility. PLoS One. (2023) 18:e0278468. doi: 10.1371/journal.pone.0278468, PMID: 36662779 PMC9858359

[48] Sun Y Tian D Zhang M Hou Y. Spatial green space accessibility in Hongkou District of Shanghai based on gaussian two-step floating catchment area method. Buildings. (2023) 13:2477. doi: 10.3390/buildings13102477

[49] Wei L Liu H Wu L. Spatial distribution of floating population in Beijing, Tianjin and Hebei region and its correlations with synergistic development. Math Biosci Eng. (2023) 20:5949–65. doi: 10.3934/mbe.2023257, PMID: 36896558

[50] Zhou C Zhou P Xuan X. Construction of community health care integration using artificial intelligence models. Aqua Water Infrastruct Ecosyst Soc. (2024) 73:688–706. doi: 10.2166/aqua.2024.038

[51] Yang J Alisan O Ma M Ozguven EE Huang W Vijayan L. Spatial accessibility analysis of emergency shelters with a consideration of sea level rise in Northwest Florida. Sustainability. (2023) 15:10263. doi: 10.3390/su151310263

[52] Zheng Y Zhu J Li J Li G Shi H. Burrowing invertebrates induce fragmentation of mariculture Styrofoam floats and formation of microplastics. J Hazard Mater. (2023) 447:130764. doi: 10.1016/j.jhazmat.2023.130764, PMID: 36682250

[53] Shi L Zhang D Zhou Y Wu Y. Work stress, perceived social support, and mental health among Chinese migrant workers: evidence from a nationwide survey. J Occup Health. (2020) 62:e1215832741028

[54] Zhang Y Wang S Luo X. Health information dissemination in social networks: evidence from Chinese migrant workers. Soc Sci Med. (2019) 240:112542

[55] Li X Chen M Chen Y. Challenges faced by rural-to-urban migrants in China: evidence from qualitative interviews. Int J Environ Res Public Health. (2021) 18:3021.33804140

[56] Xue K Yu K Zhang H. Accessibility analysis and optimization strategy of urban green space in Qingdao City Center, China. Ecol Indic. (2023) 156:111087. doi: 10.1016/j.ecolind.2023.111087

[57] Wang Z Yuan C Zhang X Liu Y Fu M Xiao J. Interannual variations of sargassum blooms in the Yellow Sea and East China Sea during 2017–2021. Harmful Algae. (2023) 126:102451. doi: 10.1016/j.hal.2023.102451, PMID: 37290886

[58] Akakba A Lahmar B. Identification and analysis of spatial access disparities related to primary healthcare in Batna City, Algeria. Geospat Health. (2023) 18. doi: 10.4081/gh.2023.1238 38112566

[59] Alisan O Ulak MB Ozguven EE Horner MW. Location selection of field hospitals amid Covid-19 considering effectiveness and fairness: a case study of Florida. Int J Disaster Risk Reduct. (2023) 93:103794. doi: 10.1016/j.ijdrr.2023.103794, PMID: 37309508 PMC10251725

[60] Barboza-Solis C Barahona-Cubillo J Fantin R. Health inequalities in the geographic distribution of dental practitioners in Costa Rica: an ecological study. Community Dent Oral Epidemiol. (2024) 52:39–46. doi: 10.1111/cdoe.12899, PMID: 37515401

[61] Chen B Tan D. Industrial robots and the employment quality of migrant Workers in the Manufacturing Industry. Sustainability. (2023) 15:7998. doi: 10.3390/su15107998

[62] Dumedah G Iddrisu S Asare C Adu-Prah S English S. Inequities in spatial access to health services in Ghanaian cities. Health Policy Plan. (2023) 38:1166–80. doi: 10.1093/heapol/czad084, PMID: 37728231

[63] Guan A Pruitt SL Henry KA Lin K Meltzer D Canchola AJ. Asian American enclaves and healthcare accessibility: an ecologic study across five states. Am J Prev Med. (2023) 65:1015–25. doi: 10.1016/j.amepre.2023.07.001, PMID: 37429388 PMC10921977

